# Why Should Implementation Science Matter in Simulation-based Health Professions Education?

**DOI:** 10.7759/cureus.3754

**Published:** 2018-12-20

**Authors:** Raluca Dubrowski, Adam Dubrowski

**Affiliations:** 1 Miscellaneous, Memorial University of Newfoundland, St. John's, CAN; 2 Emergency Medicine, Memorial University of Newfoundland, St. John's, CAN

**Keywords:** simulation, implementation, model, education

## Abstract

Simulation is a key contributor to quality medical education. However, results achieved when simulation programs are developed and tested in controlled experimental settings do not automatically translate into improved learner outcomes when these programs are implemented in real-world settings. Although over the last decade implementation science has emerged as a field intended to guide the implementation of evidence-based programs in various contexts, recent reviews suggest that it has not been integrated into simulation-based education. Implementation science is defined as a rigorous study of methods that allow for a systematic uptake of research findings and other evidence-based practices. The ultimate goal of implementation science is to provide an evidence-based approach to program delivery in practice in order to achieve the targeted health and education outcomes and maximize the return on research investments. The main reason is that in order to achieve the intended outcomes, it is crucial to pay attention to both program quality and implementation quality. In other words, having a good simulation program does not guarantee achieving the intended learning outcomes.

In this editorial we propose to highlight the research to practice gap in simulation-based health professions education, introduce the concept of implementation science and how it can serve to close the gap, and provide an example of a model derived entirely based on other models and frameworks existing in the field of implementation science to help simulation program directors and other administrators to implement simulation programs into educational practice.

## Editorial

Research to practice gap in simulation-based health professions education

Simulation has been established as one of the most significant areas of impact in the field of health professions education [1]. This likely relates to the emergence of simulation as part of the mainstream health professions education, as well as an increasing body of evidence demonstrating its effectiveness. For instance, a recent review of research in simulation shows that it works in several key areas: providing deliberate practice as a supplement to clinical teaching; yielding cohorts of homogeneous learners with similar skill levels through the use of mastery-learning approaches; and, perhaps most importantly, improving patient outcomes by transferring skills to patient care [1].

However, the results achieved when simulation programs are developed and tested in controlled experimental laboratory settings (i.e., educational research) may not automatically translate into improved learner and patient outcomes when these programs are implemented in real-world settings (i.e., educational practice) [1]. This is due to the fact that the implementation context influences how implementation unfolds and the outcomes achieved. For instance, the infrastructure and human resources are different in research-focussed academic centres and rural teaching hospitals. As a result, programs that rely on computerized mannequins and highly trained educators to run them and debrief the training sessions, or have the appropriate staff off-time to implement educational sessions will likely have low implementation success in rural teaching hospitals where such equipment is not available and staff clinicians are not trained to provide proper debriefing. This means that the program needs to be altered, or efforts need to be made to increase readiness for implementation. Therefore, the potential of simulation is not always being fully realized. In this editorial, we argue that implementation science can provide a solution to the research to practice gap because of its demonstrated effectiveness in building implementation capacity and increasing implementation quality.

Implementation science as means to close the gap

Over the last decade, the field of implementation science has evolved as a means to reduce the research to practice gap with the goal of improving health and educational outcomes and maximizing the return on research investments. Implementation science is defined as “the scientific study of methods to promote the systematic uptake of research findings and other evidence-based practices” [2]. It provides guidance in the form of theories, tools, resources, strategies, and research designs for how to implement a program, what outcomes to measure along the way, how to pay attention to the implementation context, and how to report implementation findings (e.g., detailed description of the program and the implementation context).

It is important to note that, arguably, program implementers routinely consider various implementation elements when delivering simulation programs. For instance, most implementation efforts focus on staff training but less attention is typically given to building implementation capacity or to the implementation context and how it influences program success. As noted in a recent review of surgical simulation [3], what is missing is a systematic approach to implementation as well as comprehensive reporting details. A systematic approach to implementation means going beyond the program itself and, additionally, considering, monitoring, and measuring various aspects of the program implementation such as staff competence and self-efficacy, training and coaching, fidelity, available resources, relevant policies and incentives, organizational culture, the larger educational context, etc. Following a systematic, evidence-based implementation approach is essential given that the alternative, implementing programs in a haphazard way, is costly and leads to suboptimal outcomes. Thus, here we argue that the field of simulation-based health professions education would benefit from using implementation science to inform program implementation and achieve maximum impact.

Why use implementation science guidance to inform simulation-based health professions education?

First, program “implementation matters”, even when delivering proven programs. In a review of over 500 implementation studies, better-implemented programs yielded mean effect sizes that were two to three times larger than poorly implemented programs. Similarly, evidence shows that programs that are implemented by a well-defined implementation team, a critical component of any implementation plan, take about three years to be fully implemented with a success rate of 80%. In contrast, programs that are implemented without proper implementation teams take about 17 years to implement with an average success rate of 14% [4]. These examples illustrate that desired outcomes require more than effective programs; programs must also be well implemented.

Second, recognizing that not paying attention to implementation is costly, most major funders and research agencies (e.g., National Institutes for Health, United States of America; National Institute for Health Research, United Kingdom; Canadian Institutes of Health, Canada) started to place significant emphasis on quality implementation. This led to the move of various sectors such as education, mental health, prevention and promotion, and nursing toward using guidance from implementation science to strengthen the implementation capacity and quality of their programs (for examples, see [5]).

Third, the need for simulation-based health professions education to turn to implementation science for guidance has been recognized by the health professions education field itself in a recent paper proposing increased attention to implementation science as one of the four key ways of advancing and enriching the field [1]. This proposal aligns with findings of a recent review of simulation training in surgical residency curriculum showing a need for implementation guidance in the form of best implementation practices as well as reporting guidelines (i.e., studies that describe the nature of simulation training often fail to comment on the implementation strategies used) [3].

A quick look at a guide for how to use implementation science in simulation-based health professions education

To catalyze the influx of implementation science into simulation-based health professions education, Dubrowski et al. proposed the Adapted Implementation Model for Simulation (AIM-SIM) [5]. AIM-SIM was conceived as a practical implementation guide and a starting point for using implementation science methods in simulation-based health professions education. It includes three phases: a) stakeholder engagement and context exploration, b) pre-implementation planning, and c) program implementation with monitoring and ongoing evaluation. Within each phase, a dedicated implementation team facilitates the execution of several pre-planned tasks to build implementation capacity (e.g., training; coaching; readiness for implementation), collects data capturing the implementation process and its context (e.g., measuring implementation fidelity using various procedures), and makes data-informed decisions regarding the course of the implementation (e.g., adjust training and coaching if fidelity is low). This approach results in a more thorough understanding of why and how the program worked; or, if it did not work as expected, it helps to determine whether it was due to the program itself, or to the quality of the implementation.

Summary

In this editorial, we attempt to open a long-anticipated discussion between two scientific communities - implementation science and simulation-based health professions education - about using implementation science methods to address some of the questions that many program implementers ask: Why do some programs succeed and others do not? Can we predict which ones will be successful before implementation or during early implementation stages? What are the key implementation steps that need to be followed and the contextual factors that shape their implementation? Ultimately, the goal is to increase the implementation capacity in simulation-based health professions education by offering a systematic approach to program implementation and thus stimulate a shift from letting implementation happen to making it happen.
